# *In Vitro* Morphological Assessment of Apoptosis Induced by Antiproliferative Constituents from the Rhizomes of *Curcuma zedoaria*

**DOI:** 10.1155/2013/257108

**Published:** 2013-05-20

**Authors:** Syarifah Nur Syed Abdul Rahman, Norhanom Abdul Wahab, Sri Nurestri Abd Malek

**Affiliations:** ^1^Institute of Biological Sciences, Faculty of Science, University of Malaya, 50603 Kuala Lumpur, Malaysia; ^2^Centre for Foundation Studies, University of Malaya, 50603 Kuala Lumpur, Malaysia

## Abstract

Bioassay-guided isolation of the active hexane fractions of *Curcuma zedoaria* led to the identification of five pure compounds, namely, curzerenone (**1**), neocurdione (**2**), curdione (**3**), alismol (**4**), and zederone (**5**) and a mixture of sterols, namely, campesterol (**6**), stigmasterol (**7**), and **β**-sitosterol (**8**). Alismol has never been reported to be present in *Curcuma zedoaria*. All isolated compounds except (**3**) were evaluated for their cytotoxic activity against MCF-7, Ca Ski, and HCT-116 cancer cell lines and noncancer human fibroblast cell line (MRC-5) using neutral red cytotoxicity assay. Curzerenone and alismol significantly inhibited cell proliferation in human cancer cell lines MCF-7, Ca Ski, and HCT-116 in a dose-dependent manner. Cytological observations by an inverted phase contrast microscope and Hoechst 33342/PI dual-staining assay showed typical apoptotic morphology of cancer cells upon treatment with curzerenone and alismol. Both compounds induce apoptosis through the activation of caspase-3. It can thus be suggested that curzerenone and alismol are modulated by apoptosis via caspase-3 signalling pathway. The findings of the present study support the use of *Curcuma zedoaria* rhizomes in traditional medicine for the treatment of cancer-related diseases. Thus, two naturally occurring sesquiterpenoids, curzerenone and alismol, hold great promise for use in chemopreventive and chemotherapeutic strategies.

## 1. Introduction

Cancer is a major cause of death worldwide. Surgery, chemotherapy, and other additional cancer treatment have been known to reduce only 5% of cancer-related death and its side effects. Thus, lots of research is now being carried out in searching for better chemotherapy drug from naturally occurring compounds which can suppress or prevent the process of carcinogenesis [1, 2].

Members of the Zingiberaceae family are widely used as spices, food preservative, cooking ingredients, and also as alternative medicine in some countries, especially in the Asian region. The practice of using gingers in Asian cooking and food may be the main reason of lower incidence of cancer-related death (such as colon and breast cancers) in comparison to those found in developed western countries [3–5].

*Curcuma zedoaria* (Berg.) Rosc., locally known as “temu putih,” is a ginger species widely distributed throughout Asia including Malaysia. It is regularly consumed as spices, flavouring, in food preparation during confinement, and as an ingredient in traditional medicine to treat various ailments including cancer [6, 7]. In traditional medicine, the rhizomes are consumed either on their own or in the form of  “jamu” (a concoction brewed from fresh gingers and other ingredients).

The anticancer and biological properties of *Curcuma zedoaria* rhizomes have been extensively reported by Lobo et al. [8]. However, there are very few reports on the active components responsible for the cytotoxic effects, even less on the underlying mechanism of cell death elicited by the active components [9]. It is highly desirable to have compounds that can cause cancer cell death via apoptosis. Apoptosis eliminates malignant or cancer cells without damaging normal cells and surrounding tissues [10]. Apoptosis is characterised by cell morphological changes, chromatin condensation, DNA cleavage, and nuclear fragmentation. There are two main apoptotic pathways—the intrinsic or mitochondrial pathway and the extrinsic pathway which involves ligand binding to a death receptor, where both pathways subsequently cause activation of the caspase cascade which then trigger an ordered series of biochemical events that lead to cell changes (morphology) and death [11]. Typical morphological features of apoptotic cells can be observed through microscopic studies such as those using the inverted phase contrast and fluorescence microscope. Other features such as chromatin condensation and nuclear fragmentation can be better observed through the double staining with Hoechst 33342 and propidium iodide using fluorescence microscopic analysis. This is a convenient and rapid assay, widely used to identify live and dead cells. Hoechst 33342 is a blue fluorescing dye that stains chromatin DNA. The red fluorescing dye propidium iodide is only permeable to dead cells and cannot enter the intact plasma membrane of living cells. Thus, the staining pattern which resulted from the simultaneous use of these dyes makes it possible to distinguish normal, apoptotic, and dead cells population by fluorescence microscopy [12–15].

In the present study, attempts were made to isolate and identify the active principles from the rhizomes of *Curcuma zedoaria* collected from Yogyakarta, Indonesia. The crude and fractionated extracts from the rhizomes of *Curcuma zedoaria* were evaluated for their cytotoxicity and antiproliferative activity on six human cancer cell lines, namely, the nasopharyngeal carcinoma cell line (KB), cervical carcinoma cell line (Ca Ski), hormone-dependent breast cancer cell line (MCF-7), colon carcinoma cell line (HCT-116), lung carcinoma cell line (A549), and noncancer human lung fibroblast cell line (MRC-5) using an *in vitro* neutral red cytotoxicity assay. The presence of apoptosis induced by two active components isolated from *Curcuma zedoaria* was then investigated through morphological observation of the cancer cells using inverted phase contrast and fluorescence microscope (Hoechst 33342/PI) and the activation of caspase-3 activity.

## 2. Methods

### 2.1. General

TLC: Merck precoated plates (silica gel 60 F254) of 20.25 mm thickness. HPTLC: Merck precoated plates (silica gel 60 F254). HPLC analysis: Waters Delta Prep system equipped with a Water Prep LC controller, quaternary pump, a vacuum degasser, and UV detector (Waters 2487, Dual *λ* Absorbance Detector). The column used was a Chromolith SemiPrep RP18 endcapped 100–10 mm. NMR analysis: JEOL 400 MHz FT NMR spectrometer at 400 MHz for 1H-NMR and at 100.40 MHz for ^13^C-NMR. Internal standard used in ^1^H-NMR and ^13^C-NMR spectra CDCl_3_ (*δ*: 77.0). GC-MS analysis: GC-MS analysis was performed using an Agilent Technologies 6980 N gas chromatography equipped with a 5979 Mass Selective Detector (70 eV direct inlet); column used was HP-5 ms capillary column (5% phenylmethylsiloxane) (30.0 m × 25 mm × 25 *μ*m) initially set at 100°C, then increased to 300°C at 3°C per minute, and then held for 10 minutes using helium as carrier gas at flow rate of 1 mL min^−1^. The total ion chromatogram obtained was autointegrated by ChemStation and the components were identified by comparison with the accompanying spectral database (NIST 05, Mass Spectral Library, USA) whenever possible. Analytical grade solvents were purchased from Merck.

### 2.2. Plant Material

The rhizomes of *Curcuma zedoaria* were collected from Yogyakarta, Indonesia. A voucher specimen (herbarium number: SNM1) was deposited at the Institute of Biological Sciences, Faculty of Science, University of Malaya, Malaysia.

### 2.3. Preparation of Extracts

The oven-dried ground rhizomes (1.0 kg) were soaked in methanol (2.0 L) for three days at room temperature. The solvent-containing extract was then decanted and filtered, any traces of water were removed using anhydrous sodium sulphate, and the filtrate was evaporated under reduced pressure using a rotary evaporator to give a dark brown crude methanol extract (104.3 g, 10.4%). The methanol extract was then extracted with hexane (500 mL) and the hexane-containing extract was concentrated *in vacuo* to obtain a brown colored hexane fraction (4.6 g, 33.2% based on crude methanol extract). The hexane insoluble residue was further partitioned using ethyl acetate and water (500 mL : 500 mL) to give a dark brown ethyl acetate fraction (36.3 g, 34.8% based on crude methanol extract) and a light brown water fraction (20.8 g, 19.9%). The crude methanol (MeOH) and fractionated extracts (hexane and ethyl acetate) were dissolved in dimethyl sulfoxide (DMSO) to form stock solutions 20 mg/mL before testing. The final concentration of DMSO in test wells was not in excess of 0.5% (v/v).

### 2.4. Cell Culture

The human cancer cells KB, Ca Ski, MCF-7, HCT-116, A549, and noncancer human cell lines (MRC-5) were purchased from the American Tissue Culture Collection (ATCC, USA). The supplemented RPMI 1640 media and eagle minimum essential medium (EMEM) were purchased from Sigma-Aldrich, USA. The supplements or antibiotics such as foetal bovine serum (FBS), penicillin/streptomycin (P/S) (100 *μ*g/mL), amphotericin B (50 *μ*g/mL), and accutase were purchased from PAA Laboratories, Austria. Sodium pyruvate (11 mg/mL) purchased from Sigma-Aldrich, USA. The cells were cultured in a SHEL LAB water-jacketed CO_2_ incubator with 25 cm^3^ tissue culture flasks (Nunc, Denmark), which was observed routinely under inverted microscope (Leica DMI3000 B, Germany) for any contamination and seeded into 96-well flat bottom microtiter plate (Nunc, Denmark). The extract or pure compounds was dissolved in dimethyl sulfoxide (DMSO, Molecular Biology Grade, >99.9%) from Sigma-Aldrich, USA. Microtiter plate incubator shaker (LT Biomax 500) was used and absorbance was measured at 540 nm using an ELISA microplate reader (EMax microplate reader of Molecular Devices, USA). MCF-7 and Ca Ski were cultured in RPMI 1640 media supplemented with 10% v/v FBS, P/S, and amphotericin B and MRC-5 in EMEM media with 10% v/v FBS, P/S, amphotericin B, and sodium pyruvate. The cells were cultured in tissue culture flask in 5% CO_2_ incubator kept at 37°C in a humidified atmosphere and observed routinely under inverted microscope from any contaminations. Each fresh medium was replaced every 2 or 3 days until cell confluence was achieved and the cells were detached by using accutase.

### 2.5. Neutral Red Cytotoxicity Assay

The *in vitro* cytotoxicity activity was performed using the neutral red cytotoxicity assay based on the initial protocol as described earlier [16, 17] with some modifications. Briefly, cells were detached from the tissue culture flask with 1.0 mL solution of accutase and PBS solution. The cell pellet was obtained by centrifugation at 1,000 rpm for 5 minutes. The density of the viable cells was counted by 0.4% of trypan blue exclusion in a haemocytometer. Cells were then plated in 96-well microtiter plate (Nunc), at a concentration of 1 × 10^4^ cells/well and incubated in a CO_2_ incubator at 37°C to allow the cells to adhere. After 24 h, the cells were treated with six different concentrations, that is, 1, 10, 25, 50, 75, and 100 *μ*g/mL of each tested agent in three replicates. The plates were incubated for 72 h at 37°C in a 5% CO_2_ incubator. The untreated cells were regarded as a negative control, whilst cells incubated only with DMSO (0.5%, v/v) were used as a vehicle control. No effect due to the DMSO was observed. Doxorubicin was used as the positive control. After 72 h, the old medium was substituted with fresh medium containing 50 *μ*g/mL neutral red. The 96-well plates were placed into incubator for 3 h. This is to allow the uptake of the vital dye into the lysosomes of viable and undamaged cells. Then, the media was discarded and cells were washed with 200 *μ*L of neutral red washing solution. The dye was eluted from the cells by adding neutral red resorb solution (200 *μ*L). The plate was further incubated for 30 min with rapid agitation on a microtiter plate shaker. The optical density (OD) of the eluted dye was read at 540 nm using a microplate reader. The experiments were conducted in triplicates. The percentage of inhibition of each of the test samples was calculated according to the following formula using the OD values obtained: percentage of inhibition (%) = (OD control − OD sample)/OD control × 100%. The average of three replicates was then obtained. Cytotoxicity of each test agent is expressed as IC_50_ value. The IC_50_ value is the concentration of test agents that cause 50% inhibition or cell death, averaged from the three experiments. The IC_50_ for each extract was extrapolated from the graphs of the percentage inhibition versus concentration of test agents [17–20].

### 2.6. Isolation of Pure Compounds

Based on the result of the cytotoxic screening on the crude and fractionated extracts, it was of interest to direct further research on isolation of the components which contribute to the cytotoxic activity. The active hexane fraction was subjected to an isolation procedure to obtain compounds responsible for the bioactivity. The hexane fraction/extract (25.0 g) was loaded onto a silica gel column chromatograph eluting initially with hexane followed by hexane enriched with increasing percentages of ethyl acetate to afford 15 subfractions (subfractions 1–15). All the subfractions were then subjected to cytoxicity screening. Subractions 8 and 9 showed high cytotoxic activity on MCF-7 and Ca Ski cells. Further isolation procedure was then directed to subfractions 8 and 9. Three components were identified from subfraction 8. Alismol was isolated from subfraction 8 and identified through its mass spectral and NMR data. The remaining components of subfraction 8 were positively identified as dehydrocurdione and dihydroalismol through a detailed study of its mass spectral data. Subfraction 9 contained a mixture of sterols (campesterol, stigmasterol, and *β*-sitosterol). Curzerenone, curdione, and neocurdione were isolated from subfraction 6 of the hexane fraction. All the compounds isolated were identified by comparison of their mass spectral data (obtained through GC-MS) and NMR analysis, with those of the literature [20–26].

### 2.7. Morphological Changes Using Phase Contrast Inverted Microscope

Observation of morphological changes of apoptotic cells was performed according to the method [12] with slight modifications. Briefly, 5 × 10^5^ cells were incubated for 48 hours with or without selected compounds at concentrations of 12.5, 25, 40, and 50 *μ*g/mL in a 60 mm diameter tissue culture dishes. The medium was discarded and cells were washed once with PBS. The morphological changes of the apoptotic cells were observed using phase contrast inverted microscope (Leica DMI 3000B, Germany) at 200x magnifications.

### 2.8. Morphological Changes Using Fluorescence Microscope

Briefly, cells were grown on tissue culture dishes and treated with or without tested compounds at concentrations of 12.5, 25, 40, and 50 *μ*g/mL. After 48 hours, the cells were harvested and washed with cold PBS to a density of 5 × 10^5^ cells/mL. The cells were suspended in Hoechst 33342 solution (10 *μ*g/mL) and were incubated at 37°C in CO_2_ for 7 minutes. After incubation with Hoechst 33342, the cells were counter stained with propidium iodide (2.5 *μ*g/mL). The samples were maintained in the dark for 15 minutes. After staining, an aliquot of cell suspension was placed on a glass microscope slides. The slides were observed immediately under inverted fluorescence microscope (DM16000B, Germany) using UV/488 dual excitation and the fluorescence was measured at ~460 nm emission of Hoechst 33342 dye and >575 nm emission of propidium iodide at 630x magnification [12–15, 27]. At least 200 total target cells were counted and the numbers of each four cellular states were recorded and analyzed using fluorescence microscopy for quantification of apoptosis and necrosis. The experiment was conducted in triplicates. The percentages of apoptotic and necrotic cells were determined according to the following formula [27]:

(1) Percentage  of  Apoptotic  Cells  (%) =(LA+DA)  LN+LA+DN+DA×100,Percentage  of  Necrotic  Cells  (%) =  DNLN+LA+DN+DA×100,

where LN are live target cells with normal nuclei (Hoechst 33342/PI: blue chromatin with organized structure), LA are live cells with apoptotic nuclei (Hoechst 33342/PI: bright blue chromatin that is highly condensed or fragmented), DN are dead cells with normal nuclei (Hoechst 33342/PI: pink chromatin with organized structure), and DA are dead cells with apoptotic nuclei (Hoechst 33342/PI: bright pink chromatin that is, highly condensed or fragmented).

### 2.9. Assessment of Caspase-3 Assay

Caspases are key mediators of cell death. Caspase-3 activity assay was performed using FITC Active Caspase-3 Apoptosis Kit (BD Pharmingen). These probes bind covalently and irreversibly to the active site of the active caspase heterodimer emitting the green fluorescent signal detected by the FL-1 channel. Briefly, the cancer cells were treated with curzerenone (25, 40, and 50 *μ*g/mL) and alismol (12.5, 25, and 40 *μ*g/mL) for 48 h. The assay was performed based on the manufacturer's protocol. Cells were analyzed using BD Accuri C6 flow cytometry and BD CFlow software.

### 2.10. Statistical Analysis

All data were presented as mean ± standard deviation. All the samples were measured in triplicates. One-way analysis of variance (ANOVA) and Duncan's multiple range tests (DMRT) statistical significance was used to determine the significant differences between groups. The statistical significance was accepted at *P* < 0.05. All statistical analyses were determined using STATGRAPHICS Plus software (version 3.0, Statistical Graphics Corp., Princeton, NJ, USA). Figures of isolated compounds were drawn using ChemDraw Ultra 7.0 software (CambridgeSoft, Cambridge, USA).

## 3. Results and Discussion

### 3.1. Preliminary Cytotoxicity Screenings of the Crude and Fractionated Extracts of *Curcuma zedoaria*

Table 1 shows the results of the preliminary screening of the crude and fractionated extracts of *Curcuma zedoaria*. Complete dose-response curves were generated and IC_50_ values were calculated for these crude extracts, averaged from 3 experiments, against 5 human cancer cell lines, namely, KB, Ca Ski, MCF-7, HCT-116, A549, and a noncancer cell: MRC-5. According to the NCI (United States National Cancer Institute of Plant Screening Program), a plant extract following incubation between 48 and 72 h is generally considered to have active cytotoxic effect if it has an IC_50_ value equal to or lesser than 20 *μ*g/mL [18–21].

The crude methanol extract showed good inhibitory effect against KB and Ca Ski human cancer cell lines (IC_50_ = 14.4 ± 0.7 and 14.2 ± 1.5 *μ*g/mL, resp.) and exhibited mild-to-weak cytotoxicity activity with IC_50_ values ranging from 21.0 ± 1.6 to 68.4 ± 1.3 *μ*g/mL against other human cancer cell lines. The hexane extract of *Curcuma zedoaria* possesed good inhibitory effect against MCF-7 and Ca Ski cells (IC_50_ = 18.9 ± 0.7 *μ*g/mL and 16.4 ± 0.9 *μ*g/mL) and weak effect against HCT-116 (IC_50_ = 41.1 ± 0.9 *μ*g/mL), whilst the ethyl acetate extracts showed mild-to-weak cytotoxicity against all human cancer cell lines with IC_50_ values ranging 21.0 ± 1.0 *μ*g/mL to 57.0 ± 1.2 *μ*g/mL. The water extract did not show any cytotoxicity against any of the tested human cancer cell lines (IC_50_  >  100 *μ*g/mL). Overall, the crude and fractionated extracts of *Curcuma zedoaria* showed IC_50_ values more than 20.0 *μ*g/mL on MRC-5, indicating that the crude and fractionated extracts are not deleterious to noncancerous cells. It can be concluded that the hexane extract was selectively toxic towards KB, MCF-7, and Ca Ski cells but does not exert damage to normal cells. Thus, these results support the traditional use of this medicinal plant in treating breast and cervical cancer.

### 3.2. Chemical Characterization of Compounds **1**–**10**

**Figure 1:**
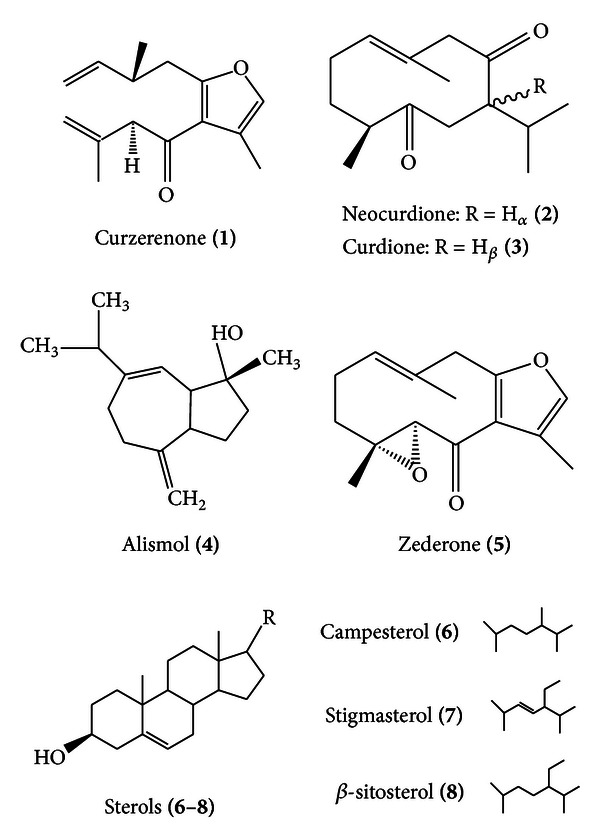
Structures of isolated compounds **1**–**8.**

Based on the above initial investigation, it was of interest to direct further research on the isolation of components that contribute to the cytotoxic activity of the plant. The hexane fraction was subjected to repeated silica gel column chromatography and HPLC to provide the isolated compounds. Bioassay-guided isolation on the active hexane fraction led to the isolation and identification of 5 pure compounds, namely, curzerenone (**1**), neocurdione (**2**), curdione (**3**), alismol (**4**), and zederone (**5**) and a mixture of sterols containing campesterol (**6**), stigmasterol (**7**), and *β*-sitosterol (**8**). Compounds **1**, **2**, and **4** were identified through their mass spectral and NMR data which were consistent with published data [20–24]. Compounds **3** and **5**–**8** were identified through their mass spectral data and consistent with published data [21–26]. Two of these components were positively identified as dehydrocurdione (**9**) and dihydroalismol (**10**) through a detailed study of its mass spectral data. Compounds **1**–**3**, **5**, and **6** have been reported for this plant, while alismol was isolated for the first time. The structures of isolated compounds **1**–**8** were illustrated in Figure 1, whilst the mass spectral and NMR data are presented below.

 Compound **1** (curzerenone): golden yellow liquid; EI-MS *m*/*z* (%): 230 (M^+^, 30), 215 (15), 162 (13), 122 (100), 94(43), 91 (17), 77 (11), 66 (11), 65 (12), 53 (8). ^1^HNMR (CDCL_3_, 400 MHz): *δ* 7.07 (1H, brs, H-11), 5.81 (1H, brs, H-5), 5.18 (1H, *t*, *J* = 1.5 Hz, H-1), 3.72 (2H, AB-system, *J* = 15 Hz, H-9a, H-9b), 2.20 (3H, d, *J* = 1.5 Hz, H-13), 1,76 (3H, d, *J* = 1.5 Hz, H-14), 1.31 (3H, s, H-15), 1.60–2.48 (4H, m, H-2 and H-3).
 Compound **2** (neocurdione): white crystals; EI-MS *m*/*z* (%): 236 (M^+^, 17), 180 (100), 167 (83), 109 (81), 69 (90), 68 (49), 67 (44), 55 (49), 41 (42). ^1^HNMR (CDCL_3_, 400 MHz): *δ* 0.92 (3H, *J* = 6.6 Hz, 14, H-3), 0.98 (3H, d, *J* = 6.8 Hz, H-12), 1.03 (3H, d, *J* = 6.8 Hz, H-13), 1.67 (3H, s, 15, H-3), 5.18 (1H, brt, *J* = 7.0 Hz).
 Compound **3** (curdione): white crystals; EI-MS *m*/*z* (%): 236 (M^+^, 17), 180 (100), 167 (83), 109 (53), 69 (52), 68 (29), 67 (30), 66 (11), 55 (33).
 Compound **4** (alismol): colorless oil; EI-MS *m*/*z* (%): 220 (M^+^, 7), 202 (29), 187 (31), 162 (40), 159 (75), 145 (37), 119 (100), 107 (38), 105 (50), 93 (48), 91 (89), 79 (39), 77 (35), 55 (33). ^1^HNMR (CDCL_3_, 400 MHz): *δ* 0.99 (3H, d, *J* = Hz, H_3_-12), 1.00 (3H, d, *J* = Hz, H_3_-13) 1.25 (3H, s, H_3_-14), 4.71, 4.76 (1H, s, H_2_-15), 5.56 (1H, s, H-6).
 Compound **5** (zederone): white crystals; EI-MS *m*/*z* (%) 246 (M^+^, 33), 231 (2), 225 (1), 219 (1), 213 (4), 203 (3), 188 (32), 175 (100), 161 (40), 147 (22), 133 (13), 119 (68), 105 (24), 91 (34), 77 (22), 65 (18), 53 (10).
 Compounds **6**–**8** (mixture containing sterols).
 Compound **9** (dehydrocurdione) EI-MS (%): 234 (M^+^, 44), 191 (27), 178 (51), 165 (87), 164 (86), 152 (81), 121 (44), 96 (60), 68 (100), 55 (30).
 Compound **10** (dihydroalismol): EI-MS (%): 222 (M^+^, 4), 204 (42), 189 (42), 175 (10), 161 (44), 147 (30), 135 (90), 121 (40), 109 (58), 95 (64), 81 (100), 71 (72), 65 (12), 55 (70).

### 3.3. Cytotoxicity Screenings of Compounds

Result from the cytotoxic activity of the isolated compounds is summarized in Table 2. All the isolated compounds except (**3**) were further tested on the selected cancer cell lines. Of these compounds, curzerenone (**1**) displayed strong cytotoxic effect against Ca Ski cell lines but moderate cytotoxicity against MCF-7 and HCT-116. In contrast, neocurdione (**2**) exhibited moderate cytotoxic activity against all cancer cell lines, whilst zederone (**5**) did not show any cytotoxic activity against the selected cell lines. This may be due to the solubility problem since zederone crystallized in the 96-well microtiter plate containing medium. However, alismol (**4**) demonstrated high cytotoxic activity against MCF-7, Ca Ski, and HCT-116. *β*-sitosterol (**8**) has been reported previously by [21] and has low cytotoxicity against MCF-7, Ca Ski, and HCT-116. This data is in good agreement with our current study; the mixture containing compounds (**6**–**8**) was not active against the selected cell lines. It is important to note that all the isolated compounds did not show toxicity towards the normal cell (MRC-5) except for curzerenone (**1**) and neocurdione (**2**). This study, therefore, showed that alismol and curzerenone contributed to the cytotoxic activity of *Curcuma zedoaria* rhizomes. Even though the pure compounds are not as effective as doxorubicin in killing the cancer cells, the isolated naturally occurring compounds (curzerenone and alismol), however, have lower cytotoxicity against the normal cells.

To our knowledge, there is no report on the cytotoxic activity of curzerenone (**1**), neocurdione (**2**), and alismol (**4**) against MCF-7, Ca Ski, and HCT-116 human cancer cell lines. Thus, this is the first report on the cytotoxic activity of the above compounds against these cell lines and alismol is reported for the first time to exist in *Curcuma zedoaria*. It is highly desirable to have a substance which is selectively active in killing cancer cells but exerted no or little damage to the normal cells [21]. If this occurs *in vivo*, then the substances will be good candidates for clinical trial.

### 3.4. Morphological Changes of Selected Human Cancer Cell Lines Using Phase Contrast Inverted Microscope

The apoptogenic property of the active compounds (curzerenone and alismol) was investigated through morphological changes in MCF-7, Ca Ski, and HCT-116 human carcinoma cells. Apoptotic cells displayed typical common features such as cell shrinkage, nuclear condensation, membrane blebbing, chromatin cleavage, and formation of pyknotic bodies of condensed chromatin [12]. These distinctive typical forms of morphological changes in apoptotic cells are widely used for the identification and quantification of apoptosis [27]. Thus, determination of the morphological changes to define apoptosis was visualised using inverted phase contrast microscope.

**Figure 2:**
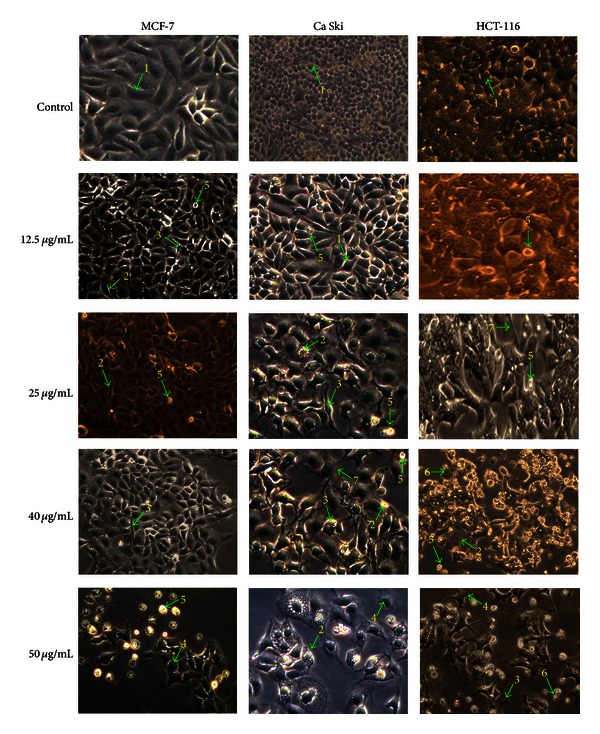
Representative photomicrograph shows morphological changes of selected cancer cells (MCF-7, Ca Ski, and HCT-116). Cells were treated with curzerenone for 48 h and imaged by inverted phase contrast microscope (magnification 200x). Arrows indicates (1) control cell, (2) condensed nuclei, (3) cell shrinkage, (4) membrane blebbing, (5) apoptotic bodies, (6) bubbling, and (7) echinoid spikes.

**Figure 3:**
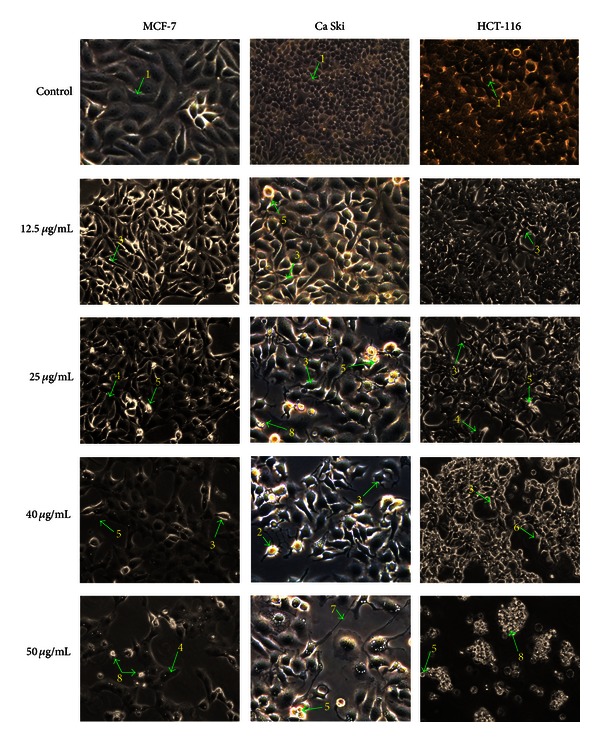
Representative photomicrograph shows morphological changes of selected cancer cells (MCF-7, Ca Ski, and HCT-116). Cells were treated with alismol for 48 h and imaged by inverted phase contrast microscope (magnification 200x). Arrows show (1) control cell, (2) condensed nuclei, (3) cell shrinkage, (4) membrane blebbing, (5) apoptotic bodies, (6) blistering, (7) echinoid spikes, and (8) pyknotic body.

After incubation with tested compounds for 48 h, morphological alterations in MCF-7, HCT-116, and Ca Ski cells were observed (Figures 2 and 3) in comparison to control cells. Visualization of the control (untreated) cells showed that the cells maintained their original morphology form containing several nucleoli. Most of the control cells were adherent to the tissue culture dishes. In contrast, exposure of MCF-7, HCT-116, and Ca Ski cancer cells treated with curzerenone and alismol for 48 h revealed typical apoptotic features such as rounding, shrinkage, membrane blebbing, and losing contact with adjacent cells.

It was observed that as the dose of the tested compounds was increased, cells undergoing apoptosis also resulted in other types of morphological changes such as echinoid spikes on the surface of apoptotic cell, apoptotic bodies, and decrement of cell number.

The apoptotic cells produced loss of cellular adhesion to the substrate and most of the cells even detached from the surface of the tissue culture dishes plate and appeared floating in the culture medium. Relatively early detachment from their basal membrane is characteristic of apoptosis of monolayer adherent cells and is called anoikis [15]. The different dose treatments applied to cancer cells induced morphological alterations of the cellular surface. Apoptosis surface morphology observed was very similar for the three cell types when treated with curzerenone and alismol. This implies that no matter what the induction pathway is, once apoptosis begins the cell is likely to follow in the same morphological alterations. This is consistent with the well-known highly conserved nature of apoptosis.

Cell death via necrosis was also visible through inverted phase contrast microscope. The increasing dose of alismol and curzerenone cause the cells to undergo necrosis. Cell death through necrosis was scored on the basis of typical morphological alteration affecting the plasma membrane [28]. The necrotic cells can be seen in the treated human cancer cells treated especially with higher dose of alismol. Necrotic cells such as bubbling is clearly visible when cells were treated with 40 and 50 *μ*g/mL of alismol on HCT-116 cells. The loss of control of the water influx through the plasma membrane which do not cause disruption of the membrane is known as evaginations [28]. The surface changes into a big, single bubble and eventually it is detached from the cells and the cell body remains tightly to the substrate [28].

### 3.5. Morphological Observation of Selected Human Cancer Cell Lines Using Fluorescence Microscope

In the present study, apoptosis was also assayed cytologically using the double staining of Hoechst 33342 and propidium iodide. Morphological changes in cell nuclei were determined by fluorescence microscope. The blue fluorescent Hoechst 33342 is a cell permeable nucleic acid dye usually used to identify chromatin condensation and fragmentation by staining the condensed nuclei of apoptotic cells. The red-fluorescent propidium iodide is a cell impairment DNA-binding dye, which can only stain the cells in situations where there is increase in plasma membrane permeability and loss of plasma membrane integrity.

**Figure 4:**
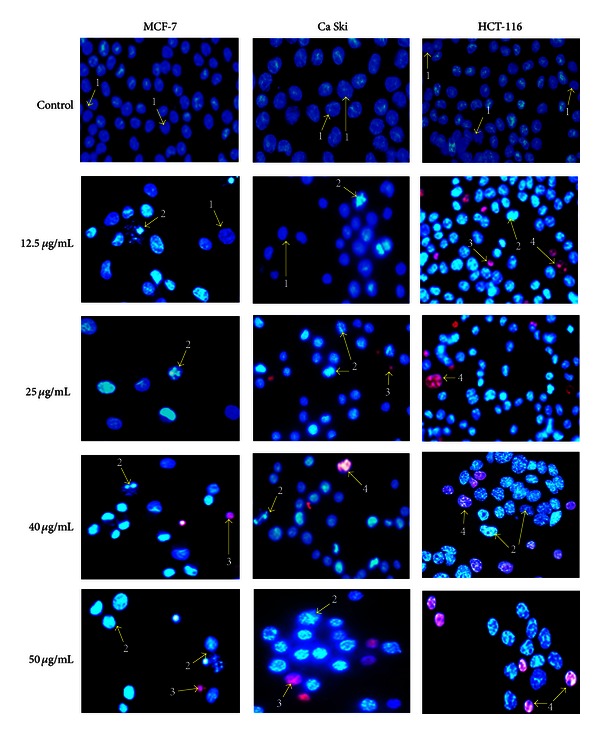
Representative composite images show morphological changes of selected cancer cells (MCF-7, Ca Ski, and HCT-116) detected with dual staining of Hoechst 33342/PI. Cells were treated with curzerenone for 48 h and imaged by fluorescence microscope (magnification 630x). Arrows indicate (1) viable cells with normal nuclei, (2) live cells with apoptotic nuclei, (3) dead cells with normal nuclei, and (4) dead cells with apoptotic nuclei.

**Figure 5:**
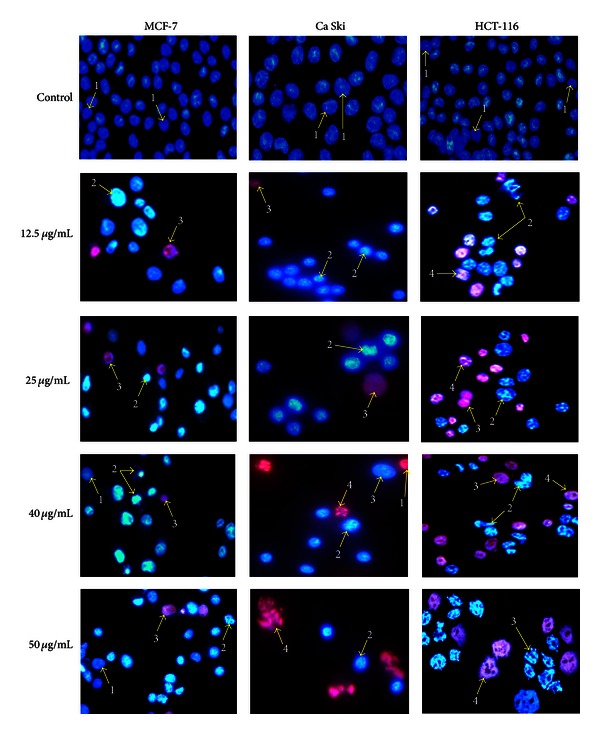
Representative composite images show morphological changes of selected cancer cells (MCF-7, Ca Ski, and HCT-116) detected with dual staining of Hoechst 33342/PI. Cells were treated with alismol for 48 h and imaged by fluorescence microscope (magnification 630x). Arrows indicate (1) viable cells with normal nuclei, (2) live cells with apoptotic nuclei, (3) dead cells with normal nuclei, and (4) dead cells with apoptotic nuclei.

The morphological observation in the cell nuclei of MCF-7, HCT-116, and Ca Ski cells for 48 h after treatment with or without tested compounds showed significant morphological alterations when compared to untreated control. As observed in Figures 4 and 5, the control or untreated cells appeared to be intact oval shape and the nuclei were stained with a less bright blue fluorescence (due to the Hoechst 33342 dye), and the absence of red fluorescence (due to the propidium iodide dye) also denoted regular, intact cells. Cells treated with tested compounds exhibited typical features of apoptosis such as cell shrinkage, chromatin condensation, and fragmentation in multiple, segregated bodies, formation of apoptotic bodies, and cell decrement. The apoptotic nuclei clearly showed highly condensed or fragmented chromatin that was uniformly fluorescent.

This can be seen in the appearance of crescents around the periphery of the nucleus or the entire chromatin was present as one or a group or featureless, bright blue spherical beads (typical detection of early apoptosis) [27]. Cells that were in late apoptosis or necrosis emit pink fluorescence. The organized structure of pink chromatin can be denoted as dead cells with normal nuclei. As membrane integrity became compromised and propidium iodide leaked into intact, even in shrunken cell, the dead cells with apoptotic nuclei emitted bright pink chromatin that is highly condensed and fragmented.

Necrotic cells are swollen and have irregular membranes and fluorescent bright pink chromatin (due to propidium iodide). The morphological alterations of necrotic cells, such as the loss of integrity of the plasma membrane [29], can be seen clearly in HCT-116 treated cells with 50 *μ*g/mL of curzerenone and alismol.

### 3.6. Quantification of Apoptotic/Necrotic Cells

The percentages of apoptotic and necrotic cells were also determined. Hoechst 33342/PI double staining allowed apoptotic cells to be distinguished from necrotic cells. The microscopic fields were photographed with fluorescence microscope (Leica DM16000B, Germany). During counting, particular care was taken not to overestimate the number of apoptotic cells by incorrectly counting too small elements corresponding to apoptotic bodies [15].

As observed in Tables 3 and 4, the percentage of apoptotic cells (apoptotic index) seemed to increase sharply, peaking at 50.0 ± 0.5% on HCT-116, 53.0 ± 2.2% on MCF-7, and 54.7 ± 1.8% on Ca Ski when treated with curzerenone (25, 40, and 50 *μ*g/mL resp.), whilst the necrotic cells seemed to remain moderate: 28.0 ± 0.5%, 17.8 ± 2.6%, and 16.3 ± 1.0% on HCT-116, MCF-7, and Ca Ski cells, respectively.

The percentage of apoptotic cells displayed increased to the highest peak at 55.3 ± 3.0% on HCT-116 and 53.7 ± 1.8% on Ca Ski cancer cells when treated with 25 *μ*g/mL of alismol. Whilst on MCF-7, the highest percentage of apoptotic cells (43.0 ± 1.0%) was achieved when treated with 40 *μ*g/mL of alismol. The percentage of necrotic cells seemed to increase sharply in all treated HCT-116, Ca Ski, and MCF-7 cancer cells peaking at 76.7 ± 0.8%, 60.3 ± 1.0%, and 59.0 ± 1.1% when treated with 50 *μ*g/mL of alismol.

Exposure of the tested compounds (alismol and curzerenone) triggers apoptosis in nearly 50% of all the cancer cells after 48 h. The increase in the percentage of necrotic cells at 50 *μ*g/mL of both tested compounds, identified by nucleus staining with PI, may correspond partly to a final phase of apoptosis, when the plasmic membrane become permeable [22]. Therefore, curzerenone and alismol have the capability to induce apoptotic and morphological changes in different types of cancer cell line, dose dependently.

### 3.7. Induction of Caspase-3

**Figure 6:**
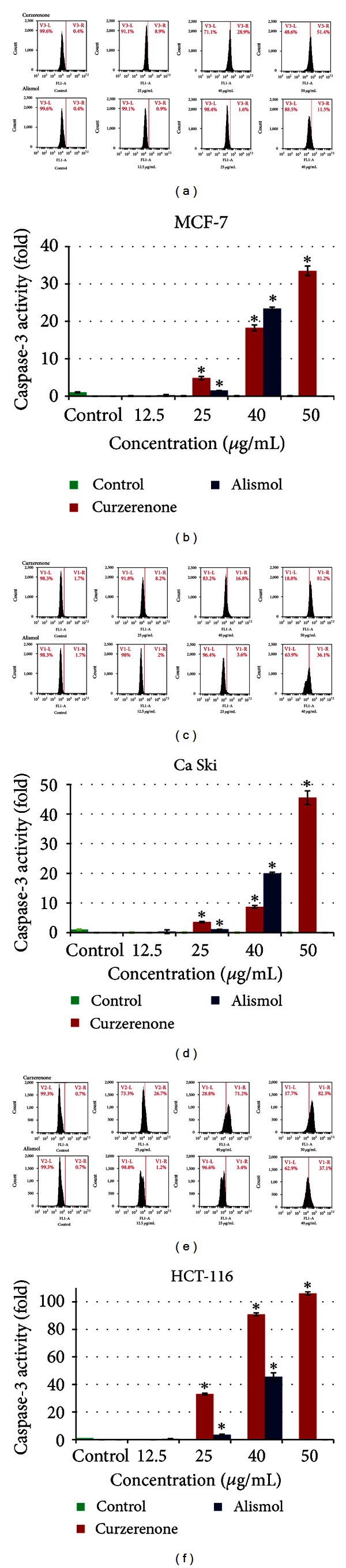
Flow cytometric analysis of apoptotic and nonapoptotic populations for active caspase-3 in (a) MCF-7, (c) Ca Ski, and (e) HCT-116. Histograms show untreated (control) cells were primarily negative for the presence of active caspase-3; whereas treated cells with curzerenone and alismol were positive for active caspase-3 (right panel). Bar chart (b, d, f) indicates caspase-3 activation as fold increase compared to control. Values were mean ± S.D from at least three independent experiments. The asterisk represented significantly different value from control (*P* < 0.05).

An *in vitro* flow cytometric assay of caspase-3 was conducted to assess apoptosis in the treated cancer cells. The apoptotic pathway in curzerenone and alismol treated MCF-7, Ca Ski, and HCT-116 cells were examined using BD Pharmigen Caspase-3 Assay Kit as described in the accompanying staining protocol. Cells were permeabilized, fixed, and stained for active caspase-3 (FITC Rabbit Anti-Caspase-3 antibody). Cells were then analysed by flow cytometry. As shown in Figures 6(a)–6(f), curzerenone and alismol significantly activated caspase-3 in a dose-dependent manner. The present study revealed that curzerenone and alismol induced significantly the elevation of caspase-3 on MCF-7, Ca Ski, and HCT-116 cells as compared to untreated cancer cells. Curzerenone exhibited the highest increment in caspase-3 activity in all cancer cells especially in HCT-116 which were 100-folds higher compared to untreated cells. The caspase-3 activity achieved the highest increment of 45-folds in HCT-116 upon treatment with 40 *μ*g/mL alismol. Cancer cell death phenomenon could be induced through different pathways. A major part of this phenomenon could be mediated by caspase-3, the promoter of apoptosis. These findings confirmed that curzerenone and alismol are capable of inducing apoptosis in MCF-7, Ca Ski, and HCT-116 cells by activation of caspase-3 and which is reported for the first time in this communication.

## 4. Conclusions

*Cucuma zedoaria* hexane extract and compounds (curzerenone and alismol) showed a profound effect on MCF-7, Ca Ski, and HCT-116 by exhibiting its cytotoxicity towards the cancer cell. In contrast, the hexane extract and compounds did not induce cytotoxic effect in MRC-5.

Morphological analysis using inverted phase contrast microscope, Hoechst 33342/PI dual staining procedures by fluorescence microscope and through activation of caspase-3, showed that curzerenone and alismol were able to trigger cell death of MCF-7, Ca Ski, and HCT-116 human cancer cells through apoptosis in a dose-dependent manner. Therefore, curzerenone and alismol, two naturally occurring sesquiterpenoids, have the capability to induce apoptosis possibly through the caspase-3 intrinsic signaling pathway.

The finding of the present study thus supports the use of *Curcuma zedoaria* rhizomes in traditional medicine for the treatment of cancer-related diseases especially breast, cervical, and colon cancers. As the rhizomes are also widely consumed as salad in food without any known undesirable side effect, it can be assumed that the plant is safe for consumption at the normal dose as food. *Curcuma zedoaria* is therefore a promising dietary agent that holds great promise for use in chemopreventive and chemotherapeutic strategies. Nevertheless, further investigations are necessary to validate its therapeutic claims and to determine the mode of action of alismol and curzerenone.

At this stage, it is not possible to ascertain the complete pathway by which cell death occurs. Further studies that provide data leading to mechanism(s) of cell death are now underway.

## Figures and Tables

**Table 1:** Cytotoxic activity (IC_50_ values (*μ*g/mL)) of crude and fractionated extracts of *Curcuma zedoaria *against human cancer and noncancer cell lines.

Extracts	IC_50_ (*μ*g/mL)					
	KB	Ca Ski	MCF-7	HCT-116	A549	MRC-5
Methanol	14.4 ± 0.7	14.2 ± 1.5	21.0 ± 1.6	68.4 ± 1.3	28.0 ± 0.9	36.5 ± 2.0
Hexane	29.0 ± 1.1	16.4 ± 0.9	18.9 ± 0.7	41.1 ± 0.9	56.0 ± 0.7	37.5 ± 1.8
Ethyl acetate	29.0 ± 0.5	21.5 ± 1.7	21.3 ± 1.1	21.0 ± 1.0	57.0 ± 1.2	63.4 ± 1.6
Water	>100	>100	>100	>100	>100	>100
∗Doxorubicin	0.27 ± 0.01	0.18 ± 0.06	0.05 ± 0.01	0.24 ± 0.04	0.58 ± 0.01	0.40 ± 0.03

*Doxorubicin was used as the positive reference compound. Tabulated values are mean ± standard deviation (SD) of three replicates.

**Table 2:** Cytotoxic activity (IC_50_ values (*μ*g/mL)) of isolated compounds against selected human cancer cell lines.

Compounds	IC_50_ (*μ*g/mL)			
	MCF-7	Ca Ski	HCT-116	MRC-5
Curzerenone **(1)**	40.0 ± 0.5	8.9 ± 0.7	53.0 ± 1.5	20.0 ± 1.2
Neocurdione **(2)**	53.5 ± 1.1	46.2 ± 1.1	64.0 ± 1.3	59.0 ± 0.4
Zederone **(5)**	>100.0	>100.0	>100.0	>100.0
Alismol **(4)**	10.0 ± 0.7	8.70 ± 1.1	9.0 ± 0.9	>100.0
Sterols **(6–8)**	>100.0	>100.0	>100.0	>100.0
*Doxorubicin	0.05 ± 0.01	0.18 ± 0.06	0.24 ± 0.04	0.40 ± 0.03

*Doxorubicin was used as the positive reference compound. Tabulated values are mean ± standard deviation (SD) of three replicates.

**Table 3:** Percentage of apoptotic cells (%).

Cell lines	Compounds	12.5 *μ*g/mL	25 *μ*g/mL	40 *μ*g/mL	50 *μ*g/mL
MCF-7	Curzerenone	4.83 ± 0.58^a^	5.83 ± 0.76^a^	53.00 ± 2.2^b^	57.33 ± 4.65^b^
	Alismol	6.17 ± 0.58^a^	9.17 ± 1.5^b^	43.00 ± 1.0^d^	29.63 ± 1.1^c^

Ca Ski	Curzerenone	4.82 ± 0.59^a^	7.89 ± 1.77^b^	39.67 ± 1.89^c^	54.67 ± 1.76^d^
	Alismol	8.83 ± 0.58^a^	53.67 ± 1.76^d^	41.67 ± 0.76^c^	32.67 ± 1.04^b^

HCT-116	Curzerenone	45.00 ± 0.5^a^	50.00 ± 0.5^b^	46.5 ± 1.44^a^	49.17 ± 1.4^b^
	Alismol	48.83 ± 1.61^b^	55.33 ± 3.01^b^	21.33 ± 2.02^a^	19.00 ± 0.87^a^

Values expressed are mean ± standard deviation (SD) of triplicate measurements. For the compounds with the different concentration, means in the same rows with different letters (a–d) were significantly different (*P* < 0.05).

**Table 4:** Percentage of necrotic cells (%).

Cell lines	Compounds	12.5 *μ*g/mL	25 *μ*g/mL	40 *μ*g/mL	50 *μ*g/mL
MCF-7	Curzerenone	2.00 ± 1.30^a^	3.83 ± 0.60^b^	5.50 ± 1.30^c^	17.83 ± 2.60^d^
	Alismol	3.83 ± 0.30^a^	5.17 ± 1.50^b^	38.33 ± 1.30^c^	58.97 ± 1.10^d^

Ca Ski	Curzerenone	3.50 ± 1.30^a^	5.57 ± 1.10^b^	11.83 ± 2.10^c^	16.33 ± 1.0^d^
	Alismol	5.83 ± 1.50^a^	14.00 ± 3.50^b^	45.33 ± 0.60^c^	60.33 ± 1.00^d^

HCT-116	Curzerenone	9.00 ± 1.50^a^	15.33 ± 0.80^b^	25.00 ± 0.50^c^	28.00 ± 0.50^d^
	Alismol	19.50 ± 1.00^a^	26.00 ± 1.30^b^	67.67 ± 2.60^c^	76.67 ± 0.80^d^

Values expressed are mean ± standard deviation (SD) of triplicate measurements. For the compounds with the different concentration, means in the same rows with different letters (a–d) were significantly different (*P* < 0.05).
